# A Fractured Inferior Vena Cava Strut Migrating to the Right Ventricle Without Any Cardiovascular Complaint: A Case Report and Review of Literature

**DOI:** 10.7759/cureus.9779

**Published:** 2020-08-16

**Authors:** Mohamed Elmassry, Gaspar Del Rio-Pertuz, Saif El-Nawaa, John Abdelmalek, Mohammad M Ansari

**Affiliations:** 1 Internal Medicine, Texas Tech University Health Sciences Center, Lubbock, USA; 2 Cardiology, Texas Tech University Health Sciences Center, Lubbock, USA

**Keywords:** inferior vena cava filter, strut, ivcf, right ventricle, pulmonary embolism, strut fracture, strut migration

## Abstract

The use of inferior vena cava filter (IVCF) as one of the last resorts for pulmonary embolism prevention has expanded over the decades. The migration of a broken strut to the right ventricle is a very unusual complication that, when present, has been associated with life-threatening events. We report a case of a 34-year-old female with an inferior vena cava (IVC) strut that migrated and was incidentally found embedded in the right ventricle without any cardiovascular signs or symptoms. This case provides evidence that such filters probably have higher rates of complications than what has been thought because those complications might remain asymptomatic.

## Introduction

Inferior vena cava filter (IVCF) is used as one of the last resorts for pulmonary embolism prevention. There is not a consensus on when exactly IVCF is indicated, but classically speaking, IVCF is indicated in the presence of an absolute contraindication or complication to therapeutic anticoagulation, or failure of anticoagulation when there is acute proximal venous thrombosis [1-8]. One of the less common long-term complications from IVCF is migration, either the filter itself or a strut [9]. Following the venous flow, the most proximal organ for migration is the right heart. Implantation on the right ventricle can progress to life-threatening events, such as tamponade and even death [9,10]. We report a case of an IVCF strut that was incidentally found implanted in the anterior wall of the right ventricle.

## Case presentation

**Figure 1:**
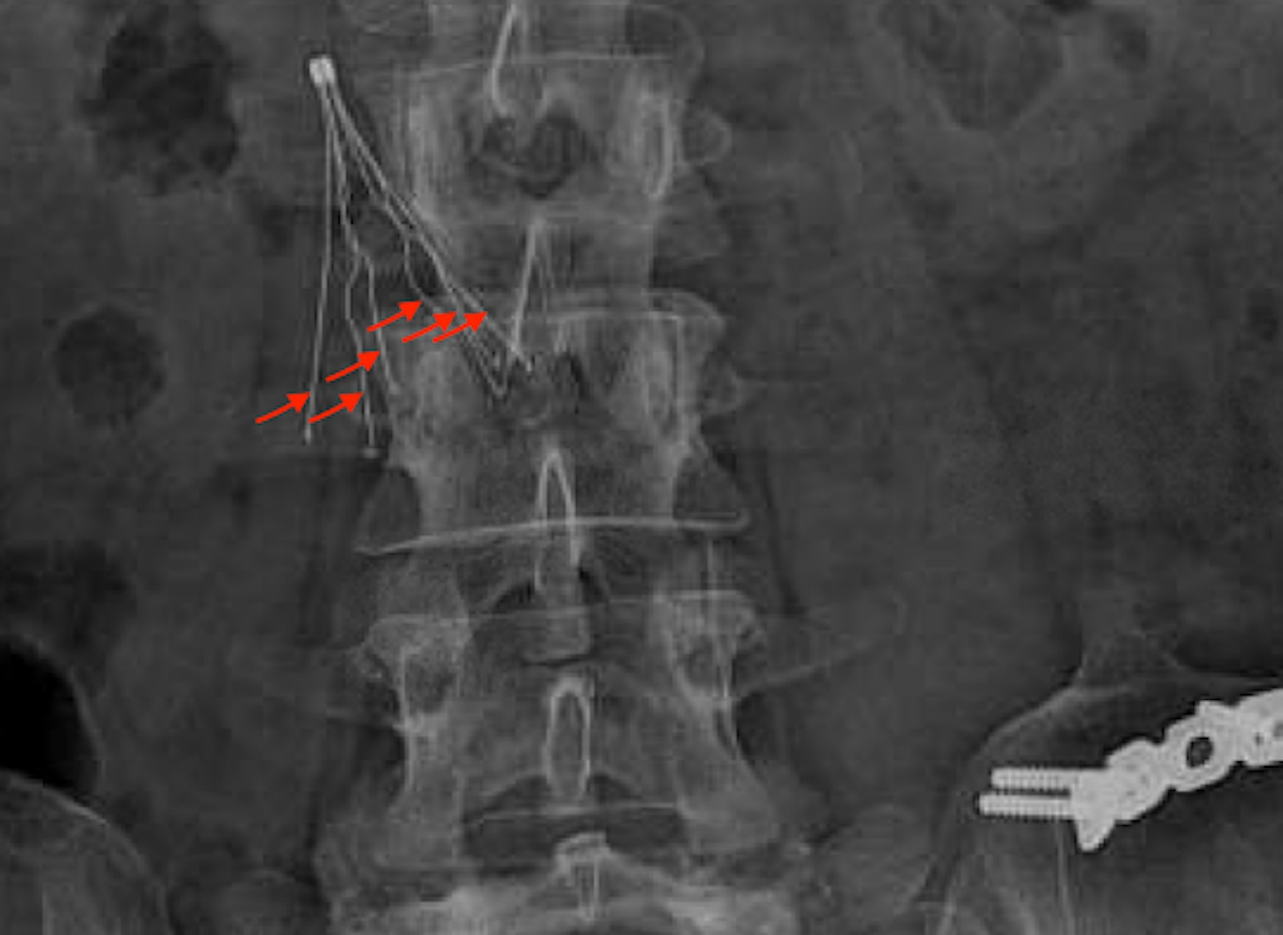
X-ray of the thoracic and lumbar spine showing the inferior vena cava filter with all six struts intact.

A 34-year-old female presented 14 years ago to an outside facility after a severe motor vehicle accident. She suffered from extensive bilateral lower extremity injuries. She underwent multiple surgeries and hardware placement in the left hip and femur. She then developed bilateral lower extremity proximal deep venous thrombosis (DVT). During hospitalization, she also developed multiple episodes of pulmonary embolism despite being on anticoagulation. The decision was made to place a Greenfield inferior vena cava (IVC) filter. Since that time she has been on oral anticoagulation. The patient had multiple clinic visits during the following years. At one visit, she presented with lower back pain, and as a part of her workup, an X-ray of the thoracic spine showed that the IVC filter was in place and the struts were intact (six struts) (Figure 1). 

**Figure 2:**
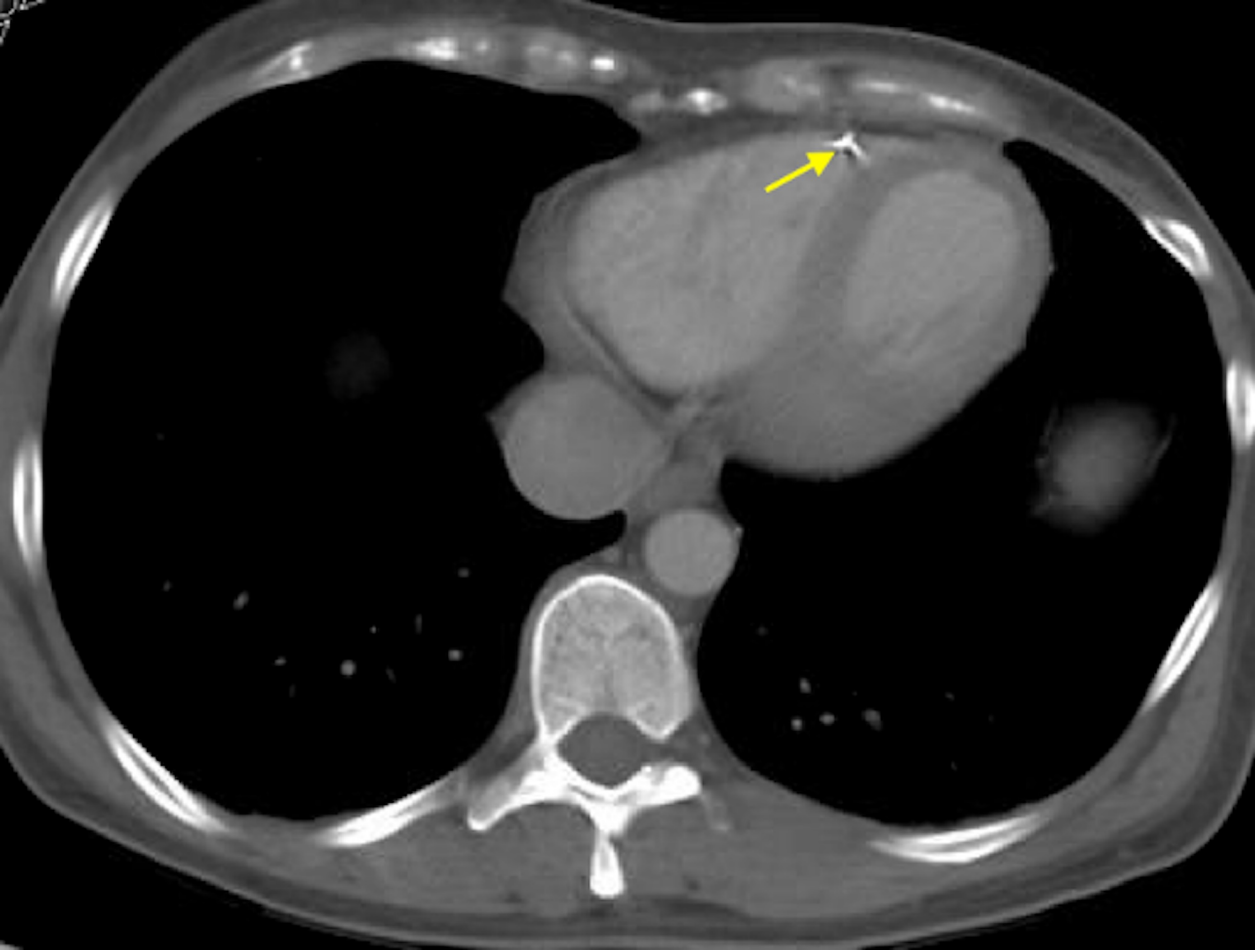
CT of the chest (axial section) showing one of the inferior vena cava filter struts embedded in the anterior wall of the right ventricle (yellow arrow).

**Figure 3:**
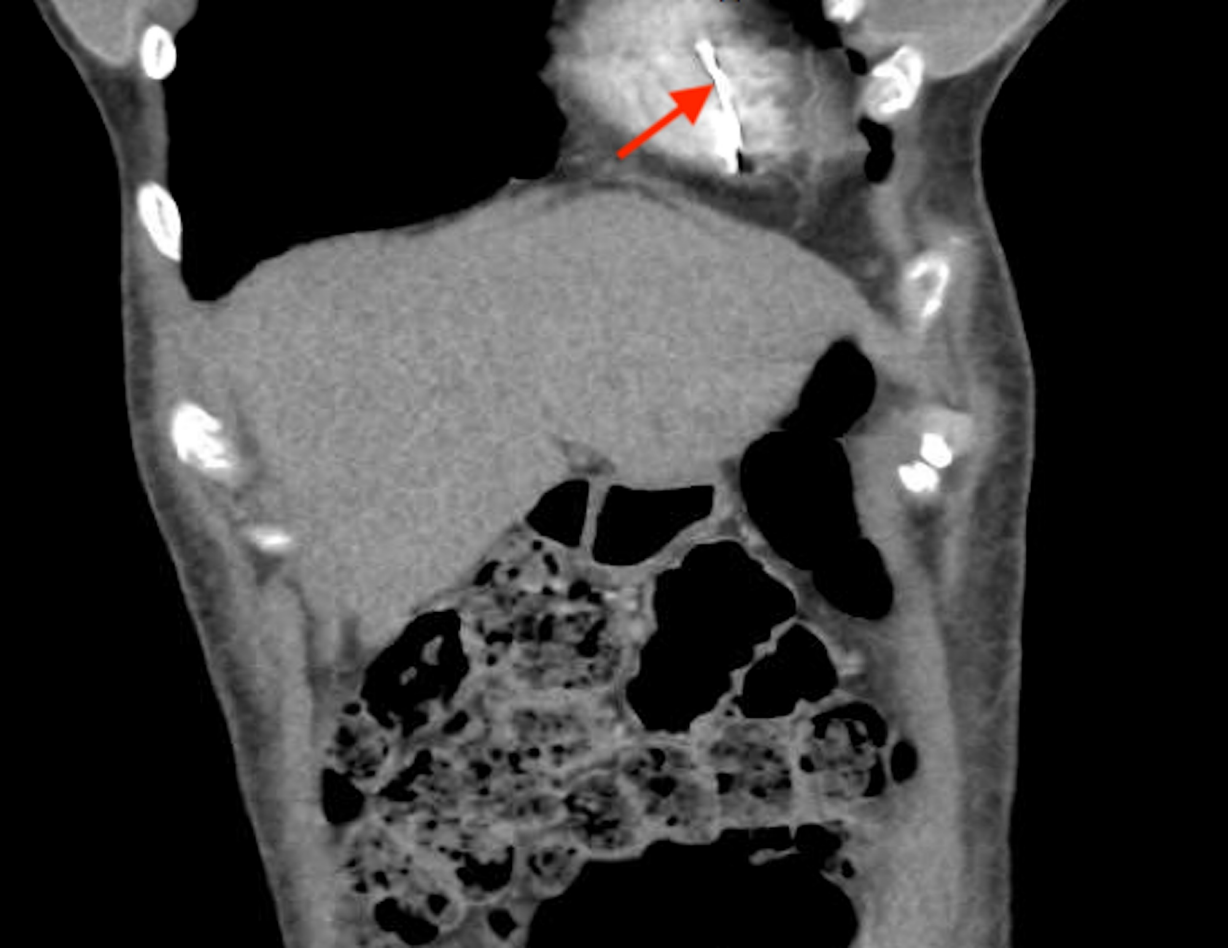
CT of the chest and abdomen (coronal section) showing the migrated inferior vena cava filter strut (red arrow).

**Figure 4:**
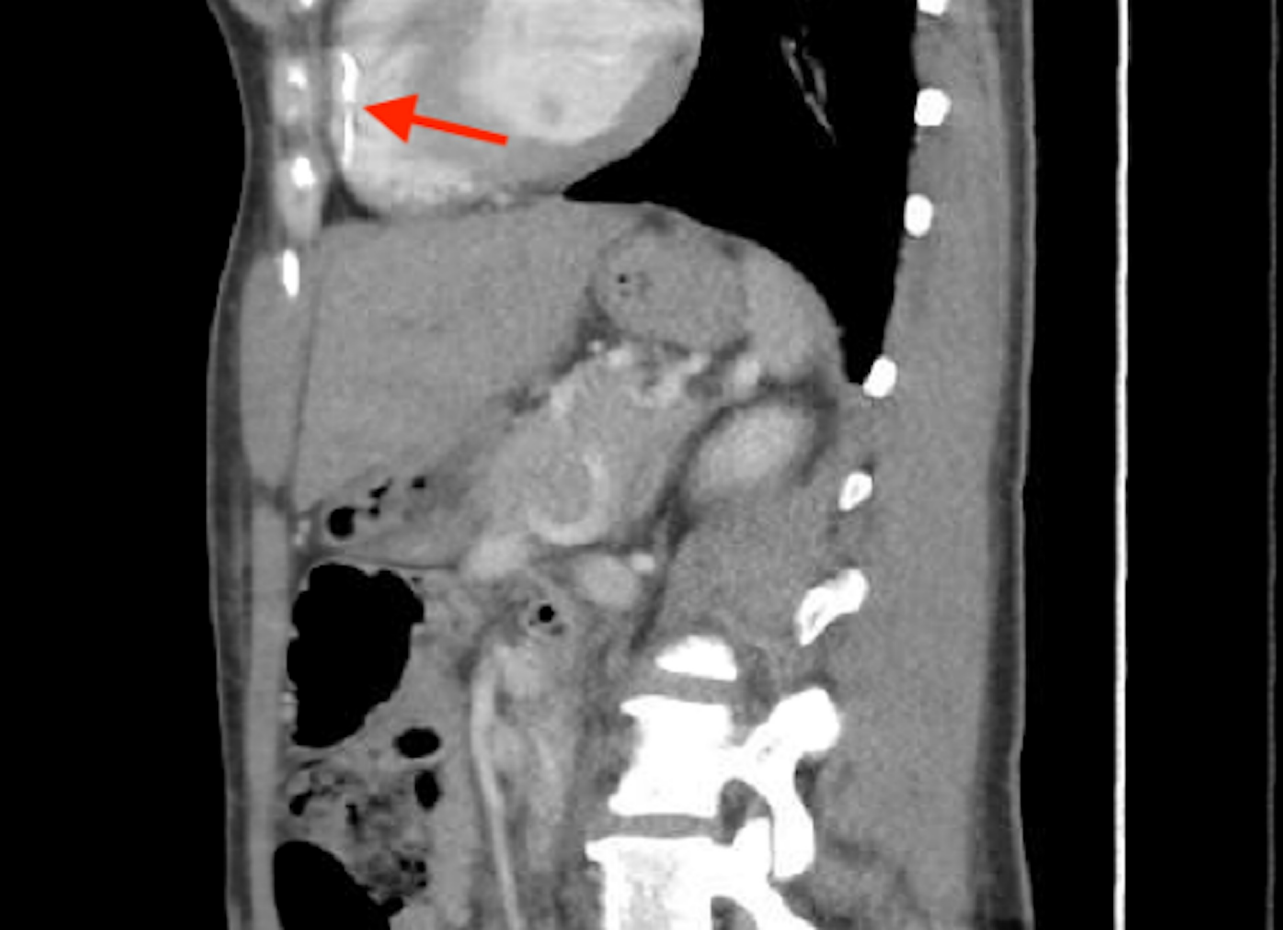
CT of the chest and abdomen (sagittal section) showing the migrated inferior vena cava filter strut (red arrow).

In March 2019, she presented to our facility with right flank pain. As part of her workup, she underwent a CT of the chest, abdomen, and pelvis with intravenous (IV) contrast that showed a high-density linear structure in the anterior wall of the right ventricle (Figures 2-4). This was likely representing a fractured IVCF strut. 

**Figure 5:**
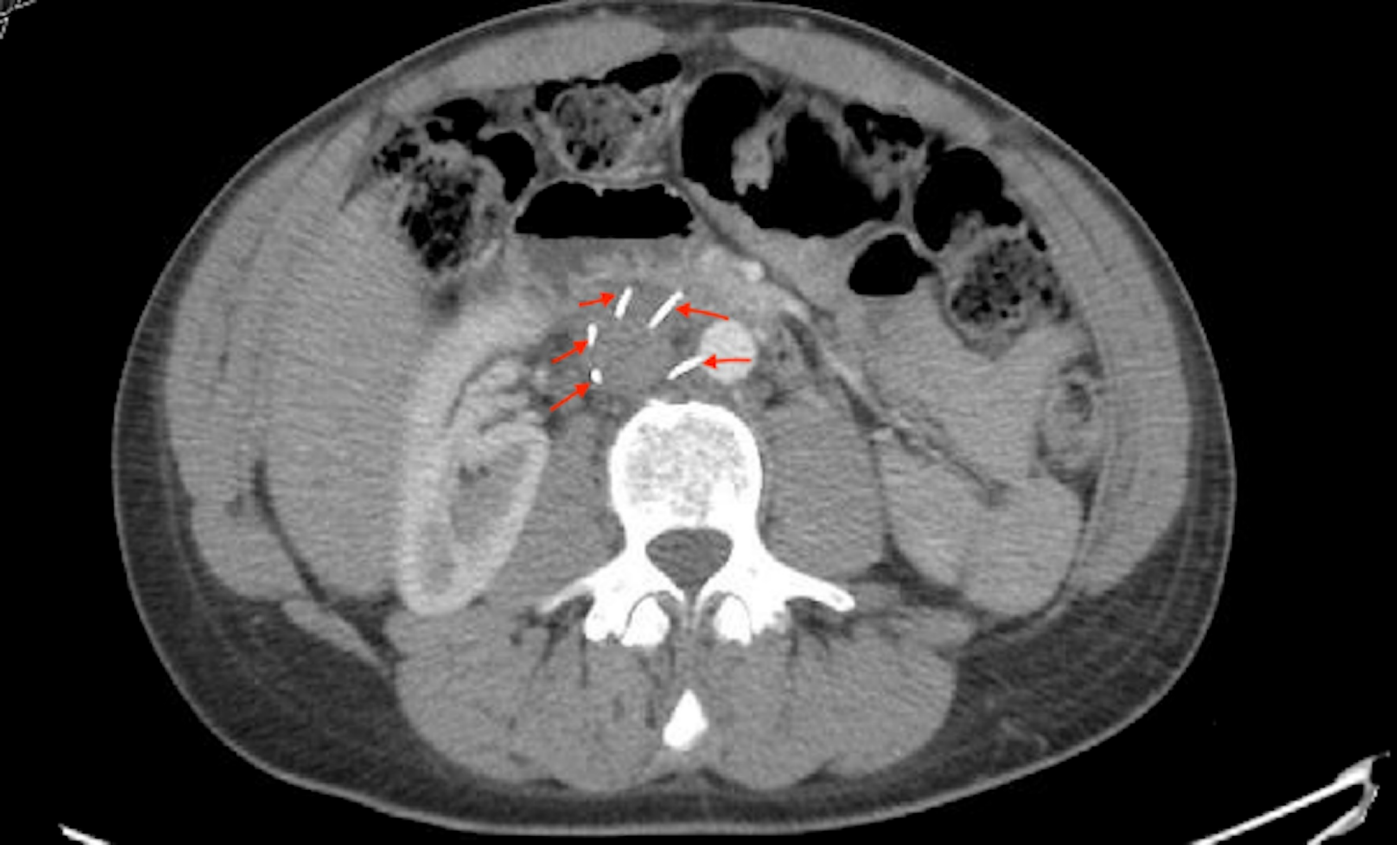
CT of the abdomen showing only five out of the six struts of the inferior vena cava filter with one strut missing.

Cardiothoracic and vascular surgery teams were consulted, and upon reviewing the CT images they agreed that the IVC filter was off-axis and missing one strut (Figure 5). They also stated that the IVC strut was likely embedded in the trabeculae of the right ventricle and endothelialized, posing minimal risk. Since the patient was asymptomatic, they decided to leave the IVC filter and the strut in place and opted for surveillance by regular follow-ups and CT scans. The patient’s right flank pain was likely musculoskeletal and responded well to the muscle relaxant. The patient was discharged home on novel oral anticoagulant with close follow-up as an outpatient. 

## Discussion

IVCFs have been used since the 1960s for the prevention of pulmonary embolism, and since then its use has expanded over the decades despite their complications [1]. After more than 50 years of experience, the current available societal guidelines on the use of IVCF are conflicting, but they all agree that that the only clear indications for an IVCF placement are when a patient had acute venous thromboembolism, in the presence of absolute contraindications to anticoagulant. One of the reasons for this uncertainty is the lack of randomized controlled trials evaluating the effectiveness of IVC and related complications [2-8].

Significant complications have been reported from IVCF, and those complications can be early during placement (e.g. allergic reaction to contrast, malposition of the filter), postprocedure (e.g. acute venous thrombosis, hematoma, arteriovenous fistula), or late after satisfactory positioning (e.g. fracture and migration of the filter or strut, chronic thrombosis) [9]. Migration and/or embolization of a fractured IVCF are rare but well-recognized complications of IVCF that have been associated with life-threatening complications [9,10]. Depending on the type of filter, the reported incidence of migration can vary from 3% to 12%, with migration to the heart being even more unlikely, ranging from 0.1% to 0.2% [11,12].

The causes of migration and fracture are multiple: failure of engagement to the venous wall, thrombus formation around the filter, trauma, IVC diameter increase due to physiologic changes, increased intra-abdominal pressure, Valsalva maneuver, coughing, or extraneous exercise [13,14]. Signs and symptoms of migration and/or embolization of the fractured IVCF depend on the organ where the filter or strut migrated. For intracardiac migration, patients can present with chest pain, hypotension, dyspnea, or any type of arrhythmia [14-16]. On the other hand, it can be either indolent, patients may not be aware that a filter fractured and migrated to an organ, like in our case where a strut from the IVCF was found as an incidental finding after cardiothoracic imaging that was done for a noncardiovascular complaint [17].

Migration of the filter or fragments into the liver, renal vein, right atrium, left and right pulmonary artery, right gonadal vein, and even into the aorta has been reported [15,18-20]. Various cases of strut and filter migration to the right ventricle have been reported recently, but all are associated with cardiovascular symptoms or outcomes ranging from a simple chest pain complaint to cardiac tamponade symptoms [16,17]. To our knowledge, this is the first reported case of a strut migrating from an IVCF that was found incidentally on the right ventricle without causing any cardiovascular symptom.

In cases of suspected migration and/or embolization of a fractured IVCF to the heart, a chest CT scan is the most suitable radiologic modality for identifying a migrated IVCF or fragments [15]. If surgery is considered, a deeper radiologic evaluation of the location and depth of penetration of the strut or filter within the heart may assist in decision making for determining if an endovascular or open surgical approach is more appropriate [16].

There are no available guidelines for the management approach of fractured and migrated struts of an IVC filter, probably due to the very low incidence of such complications as previously reported [19]. Correlation of the patient’s clinical status and location of the strut of an IVCF filter on the CT scan is key to assist in making management decisions [14,16]. A suggested conservative approach with close observation might be effective, especially with asymptomatic patients like our patient.

It should be mentioned that these complications are more frequent with longer duration of implantation [16,17,20]. Thus, we consider that the cornerstone management of this complication is prevention, by focusing on early retrieval of the filter once protection from PE is no longer needed. A multidisciplinary approach involving a cardiothoracic surgeon and an interventional cardiologist or radiologist may be indicated as proposed in our case.

## Conclusions

IVCFs have been widely used for decades for the prevention of pulmonary embolism despite their complications. IVC strut fracture and migration is a rare complication that has been reported in the literature and is usually fatal. A multimodality imaging approach is usually required to accurately visualize the site of implantation. A conservative approach with close observation might be effective, especially with asymptomatic patients.
